# Comment on Kang et al. The Effective Way of Botulinum Toxin Injection to Reduce Bite Force: Preliminary Study. *Toxins* 2025, *17*, 519

**DOI:** 10.3390/toxins18040165

**Published:** 2026-03-30

**Authors:** Elif Tarihci Cakmak, Fatma Merih Akpinar, Ekin Ilke Sen, Nalan Capan

**Affiliations:** Department of Physical Medicine and Rehabilitation, Istanbul Faculty of Medicine, Istanbul University, Istanbul 34093, Türkiye; merihcaliskan@gmail.com (F.M.A.); ekinozgorgu@gmail.com (E.I.S.); nalancapan@gmail.com (N.C.)

We read with great interest and some concerns the article by Kang and colleagues [1]. The study design contains some methodological limitations. Furthermore, we believe some aspects need to be clarified to enhance the scientific validity of the interpretations drawn from the study.

First, the title refers to “the effective way of botulinum toxin injection,” but this phrase is inappropriately assertive due to the methodological limitations of the study. In particular, the inclusion of asymptomatic participants and the reliance solely on bite force reduction as an outcome measure do not provide sufficient evidence to draw conclusions about the therapeutic efficacy of botulinum toxin type A (BoNT-A) injections. For these reasons, the term “effective” in the title cannot be justified based on the current data.

As emphasized in the literature [2,3], it is crucial to evaluate parameters such as pain, chewing efficiency and patient-reported outcomes in studies investigating the clinical importance of BoNT-A in temporomandibular joint disorders (TMDs) and bruxism. While it is predictable that applying a method whose mechanism of action is “paralysis” to the jaw-closing muscles will reduce bite force, the goal of such interventions should be to achieve functional recovery and improve the individual’s quality of life. Only holistic evaluations conducted with a biopsychosocial approach can determine whether an intervention is an “effective way”.

Moreover, the authors base their inferences for clinical practice on results obtained from “healthy male volunteers” without temporomandibular pain or dysfunction. It should be noted that due to the design of the study, drawing direct clinical relevance to these results may be misleading. Studies conducted with valid and reliable scales assessing pain, function, and quality of life in appropriate patient groups could enable conclusions to be drawn regarding BoNT-A’s therapeutic effects.

A further methodological concern is that BoNT-A applications were performed blindly in the study limits its reproducibility and casts doubt on the findings regarding the dose–response relationship. Previous studies have emphasized that ultrasound or electromyography guidance increases the accuracy of application and minimizes adverse effects, particularly in applications to deep structures such as the medial pterygoid [4]. Injections into the medial pterygoid, one of the muscles injected with BoNT-A in the study, increase the likelihood of complications such as dysphagia and dysphonia if not performed by an experienced clinician or if performed without guidance [5].

In addition to all this, it is necessary to question the requirement for healthy participants in the study to have received multi-muscle BoNT-A injections. Studies on masseter hypertrophy have shown that repeated or multiple injections cause a decrease in electromyographic activity and a decline in muscle performance without providing any therapeutic benefit or cosmetic gain [6]. Extending injections to deep muscles such as the medial pterygoid without therapeutic justification increases the risk of adverse effects when the target is only bite force reduction without any symptom reduction.

Although the authors reported using the Dental Prescale II system to measure bite force in their study, they did not provide detailed information on the occlusal site, calibration, and standardization of measurements, even though bite force is affected by all of these factors, as well as tooth morphology and fatigue [7]. In their intra-group and inter-group comparisons, conducting the evaluation using a standardized measurement is important for the consistency of the method.

Finally, the authors stated that approximately one-fifth of the participants included in the study, all of whom were in the multiple injection groups, were excluded from the analysis, which, as they also mentioned, is a significant limitation that increases the risk of bias. One way to reduce this risk would have been to perform an intention-to-treat analysis; however, it appears that they did not perform this analysis and did not provide an explanation for not doing so.

In conclusion, we emphasize that clinical science should fundamentally aim to benefit the individual. In this context, we reiterate that studies evaluating the therapeutic effects of interventions applied using standardized methods in appropriate patient groups with standardized scales can lead to “effective way” conclusions for clinical practice.

## Abbreviations

The following abbreviations are used in this manuscript:

BoNT-A	Botulinum toxin type A
TMDs	Temporomandibular joint disorders
